# Characterization of viscosupplementation formulations using chemical exchange saturation transfer (ViscoCEST)

**DOI:** 10.1186/s12967-016-0850-8

**Published:** 2016-04-12

**Authors:** Mohammad Haris, Anup Singh, Sanjana Reddy, Puneet Bagga, J. Bruce Kneeland, Fotios P. Tjoumakaris, Hari Hariharan, Francesco M. Marincola, Ravinder Reddy

**Affiliations:** Research Branch, Sidra Medical and Research Center, 26999, Doha, Qatar; Department of Radiology, Center for Magnetic Resonance and Optical Imaging, University of Pennsylvania, Philadelphia, PA USA; Center for Biomedical Engineering, Indian Institute of Technology Delhi, New Delhi, India; Department of Radiology, University of Pennsylvania, Philadelphia, PA USA; The Rothman Institute (FPT), Egg Harbor Township, NJ USA

## Abstract

**Background:**

Osteoarthritis (OA) is characterized by progressive loss of cartilage in joints, and is a major cause of pain and disability, and imposes significant health care expense. New therapies are being developed to treat the symptomatic effect of OA, one of which is intra-articular injection of viscosupplementations of different forms of hyaluronic acid (HA). The current study evaluates the chemical exchange saturation transfer (CEST) effect from two popular viscosupplementations [Hylan gf-20 (Synvisc) and hyaluronan (Orthovisc)] by targeting the exchangeable hydroxyl protons present on these molecules (ViscoCEST).

**Methods:**

ViscoCEST imaging from two viscosupplementations (Synvisc and Orthovisc) was performed on a 7T Siemens whole body MRI scanner. ViscoCEST images were collected with different combination of saturation pulse power and saturation duration. Z spectra were acquired at B_1rms_ of 3.6 μT and 1 s saturation duration by varying the frequency from −4 to +4 ppm in step size of 0.1 ppm. Field inhomogeneity (B_0_) and radiofrequency (B_1_) maps were also acquired to correct ViscoCEST contrast map for any inhomogeneity.

**Results:**

Both viscosupplementations showed broad CEST effect (ViscoCEST), which peaked ~0.8 ppm from down field of water resonance. Orthovisc showed 20 % higher ViscoCEST contrast than Synvisc suggestive of more HA component in Orthovisc. Increased ViscoCEST contrast was observed from both viscosupplementations with increase in B_1rms_ and saturation pulse duration.

**Conclusion:**

ViscoCEST has a potential to image the spatial distribution of viscosupplements in vivo in patients’ intra-articular space as well as temporal variation in their spatial distribution.

## Background

Osteoarthritis (OA), a major cause of pain and disability, is characterized by the progressive loss of cartilage tissue, increased synovial fluid, inflammation and decreased viscoelasticity of the synovial fluid within the affected joint. Hyaluronic acid (HA) is the major source of non-sulfated glycosaminoglycan (GAG) in the cartilage extracellular matrix and help in maintaining the intra-articular lubrication and optimizing the viscoelasticity of synovial fluid. In OA, the concentration and molecular weight of HA decreases in affected joints and makes it more susceptible to the mechanical stress [1, 2].

New therapies are being developed to treat the symptomatic effect of OA, one of which is intra-articular injection of viscosupplementations of different forms of HA [3, 4]. In recent years, the concept of viscosupplementation has gained widespread acceptance as a new treatment for the management of knee OA. Both preclinical and clinical studies have showed the potential of intra-articular injected HA in reducing the OA disease progression [4, 5]. In an animal model of knee OA the intra-articular injected HA reduced the cartilage degeneration and inhibited the expression of pro-inflammatory markers including interleukin-1 beta and metalloproteinase-3 [6–9]. Studies have demonstrated that the intra-articular injected HA helps with joint repair through its effect on chondrocyte growth and metabolism as well as with synthesis of endogenous HA, proteoglycan, and collagen [10]. In humans, it is observed that the intra-articular injected HA may improve cartilage morphology and reduce synovial fluid inflammation and cartilage degradation over a period of 6 months to 1 year [11, 12]. Despite various subjective studies demonstrating alleviation of patients’ pain after intra-articular injection of viscosupplementation, there is still controversy in the use and efficacy of viscosupplementations [13, 14]. Thus, a noninvasive, quantitative imaging technique that is specific to viscosupplementation is needed to probe their fate in intra-articular space following injection, and their impact on the overall GAG concentration in cartilage.

Imaging methods that have been used to assess the OA disease progression include plain X-ray and conventional magnetic resonance imaging (MRI) scans. Plain X-ray provides an indirect measure of loss or thinning of cartilage by measuring the joint space narrowing (JSN) [15]. On the other hand, MRI provides high resolution images of cartilage morphology and integrity. Changes in the cartilage thickness measurement on conventional MRI may be helpful to observe the therapeutic efficacy over a long period of potential treatment.

However, there are contradictory reports of HA treatment efficacy based on the plain-X ray and conventional MRI measurement of cartilage. Subjects either received intra-articular injection of HA or saline showed no difference for the JSN measurement [16]. Similarly, MRI study in patients with HA or saline injection found no difference for cartilage defect scoring between the two groups even after 8 weeks of treatment [17]. In another study, decreased cartilage T_2_ relaxation time has been shown after intra-articular injection of hyaluronic acid in a rat model of OA [18].

Although both techniques (plain X-rays and conventional MRI) can reflect the structural changes in cartilage, but do not have enough sensitivity to detect early biochemical changes in cartilage components. However, to date there is no in vivo study demonstrating the potential of HA viscosupplements molecules in influencing the molecular changes in articular cartilage. Recently, chemical exchange saturation transfer (CEST) imaging technique has been used to image the GAG concentration of cartilage by targeting the exchangeable hydroxyl protons (–OH) on GAG (GagCEST) [19, 20]. In GagCEST, application of frequency selective radiofrequency pulses saturates the –OH protons on GAG and exchange of this saturated magnetization with those of bulk water protons results in the decrease of the bulk water magnetization. This decrease in bulk water magnetization can be used to quantify and map the GAG concentration. Feasibility of mapping GAG in the human knee cartilage through GagCEST has been evaluated both on 3T and 7T human scanner [20]. Since hyaluronan, a non-sulfated GAG in viscosupplementations also possess –OH protons, we hypothesize that it would be possible to observe CEST effect from them (ViscoCEST), and it may be possible to probe the spatial distribution of viscosupplements in knee cartilage in vivo after intra-articular injection of viscosupplementaions.

In the current study, we evaluated the ViscoCEST effect from two popular viscosupplementations [Hylan gf-20 (Synvisc) and Hyaluronan (Orthovisc)] by exploiting the exchangeable –OH protons present on these molecules. We also assessed the saturation pulse amplitude and saturation pulse duration effect on ViscoCEST contrast of these viscosupplemantations. Finally, we compared the optimal parameters for GagCEST from endogenous GAG from cartilage and ViscoCEST from exogenous HA.

## Methods

### Theoretical

The CEST technique is based on chemical exchange between a solvent pool (typically water) and a solute pool, a small metabolite or proteins. The solute pool has exchangeable protons with a distinct chemical shift from the bulk water protons. Application of the frequency selective radiofrequency irradiation on exchangeable protons of the solute pool leads to the saturation of solute spins, with zero net magnetization, and exchange of these with bulk water protons results in the decrease of bulk water signal. This saturation transfer magnetization can be imaged to detect the CEST effect from a solute, which provides an indirect measure of solute concentration. In order for the CEST effect to be efficiently observed, the slow to intermediate exchange condition (chemical shift of exchangeable spins, *∆ω* > *k*) must be fulfilled.

In biological tissues, magnetization transfer (MT) effects from the bound water protons and the direct water saturation interfere with the CEST analysis. To account for these effects, two CEST images are acquired: one with by applying saturation pulse at the resonance frequency of the exchangeable spins and other at the equal frequency on the other side of the bulk water peak. Signal difference in these two images can alleviate the MT effect. Normalizing this difference signal with the signal obtained without saturation provides the CEST effect from the exchangeable protons of solute as given in the following equation.

$$CEST_{asym} (\Updelta \omega ) = \frac{{M_{sat} ( - \Updelta \omega ) - M_{sat} (\Updelta \omega )}}{{M_{0} }}$$

where *M*_sat_ (±∆ω) are the magnetizations obtained with saturation at ‘+’ or ‘–’ ∆ω offset of the water protons resonance and *M*_0_ is the magnetization obtained without saturation. In biological tissues due to short transfer relaxation times of water, the application of saturation pulse can lead to substantial direct water saturation which can attenuates the CEST effect. This can be minimized to some extent by using *M*_sat_ (–∆ω) [21] instead of *M*_0_ to normalize the signal differences. The other factors which can substantially affect the CEST contrast are B_0_ and B_1_ field variations. As the CEST contrast is obtained by subtracting the two images (−∆ω and ∆ω), any asymmetry generated due to the local B_0_ field inhomogeneity will contaminate the actual CEST contrast. On the other hand the B_1_ inhomogeneity affects both the CEST contrast as well as the direct water saturation. The accurate quantification and correction of B_0_ and B_1_ inhomogeneities are important to obtain reliable CEST contrast [22].

### MR imaging

For this study, we used two brands of viscosupplementations i.e. Hylan gf-20 (Synvisc, Genzyme Biosurgery) and Hyaluronan (Orthovisc, DePuy Mitek) that are being widely used for treating OA patients. Both clinical grade samples were obtained in closed plastic tubes from the manufacturer. Orthovisc has a lower molecular weight than Synvisc but contains a higher concentration of hyaluronic acid per injection than Synvisc. Both products have been already shown their treatment efficacy in alleviating pain associated with OA [23, 24] and are considered high molecular weight hyaluronic acid compounds.

The ViscoCEST imaging was performed on a 7T Siemens whole body MRI scanner (Siemens Medical Systems, Malvern, PA, USA). The experiments were performed at 37 °C using a custom designed Styrofoam chamber to maintain the temperature at 37 ± 1 °C during the course of experiment. A frequency selective saturation pulse followed by a segmented RF spoiled gradient echo (GRE) readout sequence was used. A saturation pulse train consisting of 5–30 Hanning windowed rectangular pulses of 100 ms duration each with a 200 μs delay between them was employed. The saturation pulse excitation bandwidth was 5 Hz for a 1 s pulse with 1 % bandwidth of 20 Hz. The total repetition time of the sequence was adjusted to stay within specific absorption rate (SAR) limits. The sequence parameters were: slice thickness = 10 mm, GRE flip angle = 10^o^, GRE readout TR = 5.6 ms, TE = 2.7 ms, field of view = 100 × 100 mm^2^, matrix size = 192 × 192, and one saturation pulse and 64 segments acquired every 10 s. ViscoCEST images were collected with different combination of saturation pulse power (B_1rms_ = 0.7 μT, 1.4 μT, 2.2 μT, 2.9 μT, 3.6 μT, 4.4 μT) and saturation duration. Z spectra were collected at B_1rms_ 3.6 μT and 1 s saturation duration by varying the frequency from −4 to +4 ppm in step size of 0.1 ppm. The field inhomogeneity (B_0_) and radiofrequency (B_1_) maps were also gathered using the method described in details elsewhere [20].

For reproducibility of GagCEST result at 7T as shown previously [20], we also performed GagCEST imaging from knee cartilage of a normal human healthy volunteer. The Institutional Review Board Committee of the University of Pennsylvania approved the study protocols. With informed consent the volunteer underwent GagCEST imaging using the optimal CEST parameters: B_1rms_ of 2.2 μT and 0.5 s saturation duration [20]. The other imaging parameters were-slice thickness = 5 mm, flip angle = 10°, readout TR = 5.6 ms, TE = 2.7 ms, field of view = 140 × 140 mm^2^, matrix size = 192 × 192 with delay of 8 s.

### Image processing

The acquired CEST weighted images were first corrected for B_0_ and then used to compute the CEST contrast map using equation [1]. The CEST contrast map was further corrected for any B_1_ inhomogeneity and overlaid on anatomical proton weighted image as described in detail by Singh et al. [20].

## Results and discussion

**Fig. 1:**
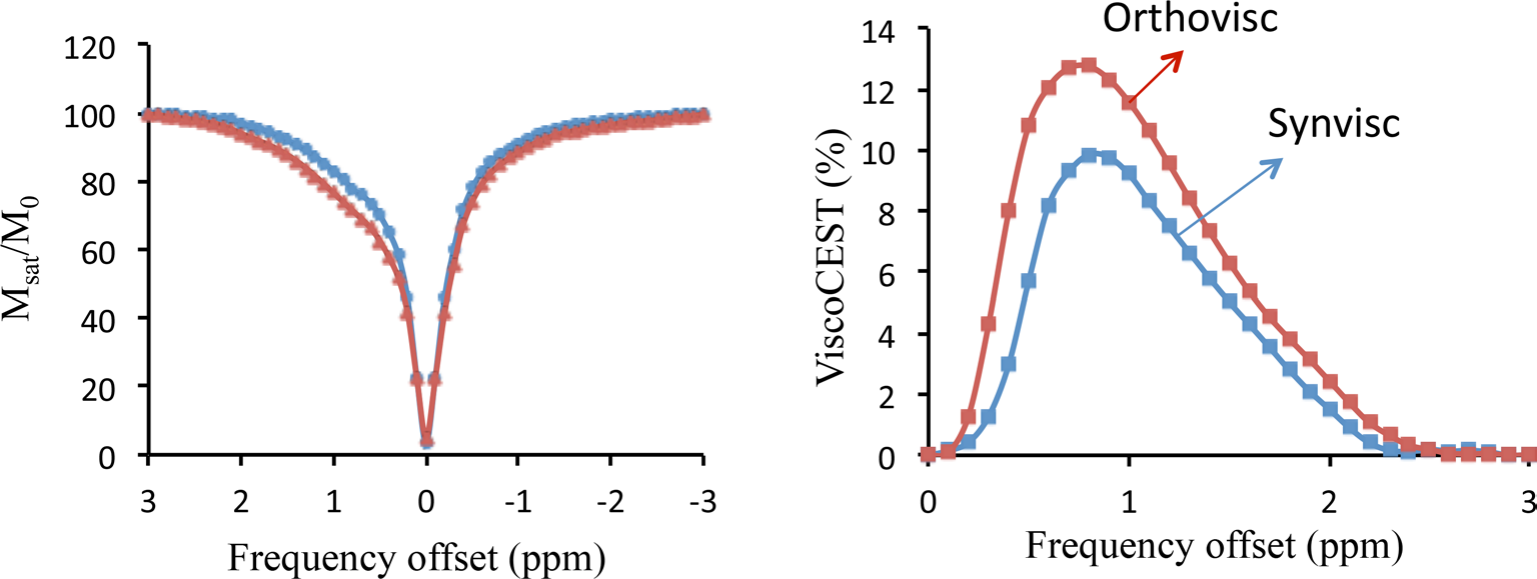
Z-spectra and Z-spectra assymetry curves show the gagCEST effect from Orthovisc and Synvisc, which peaks ~0.8 ppm. Higher gagCEST contrast is depicted from Orthovisc than Synvisc

**Fig. 2:**
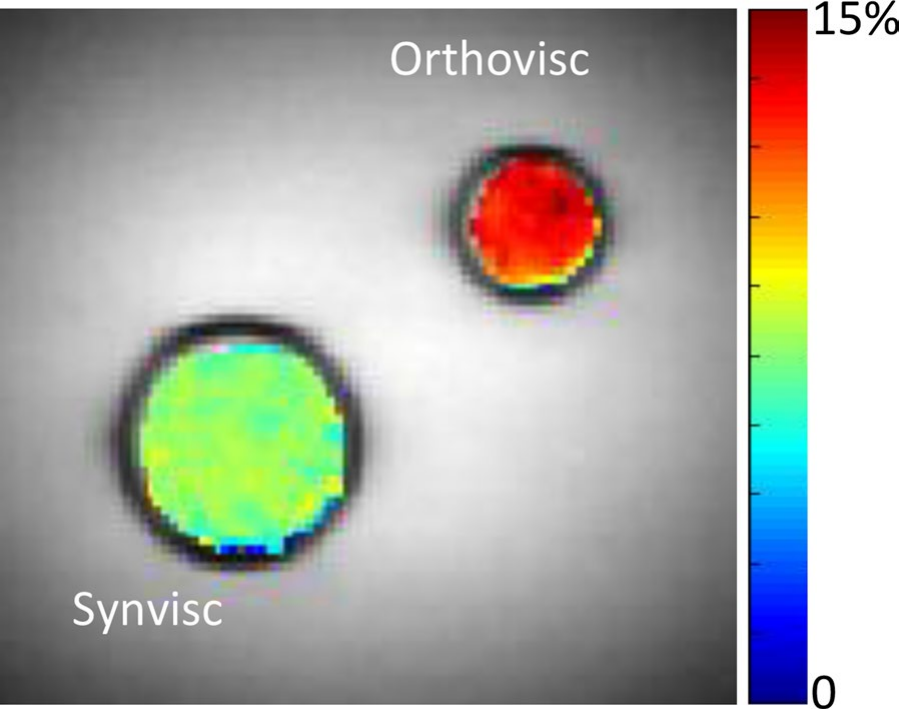
Shows the gagCEST contrast maps from Orthovisc and Synvisc overlaid on anatomical proton image. The gagCEST map is showing higher contrast from Orthovisc

Z spectra and Z spectral asymmetry curves of ViscoCEST are shown in Fig. 1. Z spectral asymmetry curves clearly showed the broad CEST peak centered ~0.8 ppm in both viscosupplementations (Fig. 1). For the same saturation parameters Orthovisc depicted higher ViscoCEST contrast compared to Synvisc as clearly observed from Z spectra asymmetry curves (Fig. 1). The ViscoCEST map at B_1rms_ of 3.6 μT and 1 s saturation duration (Fig. 2) showed ~20 % higher ViscoCEST contrast from Orthovisc than Synvisc. The higher ViscoCEST contrast from Orthovisc might be due to its higher concentration of HA and therefore possesses more exchangeable –OH protons than Synvisc.

**Fig. 3:**
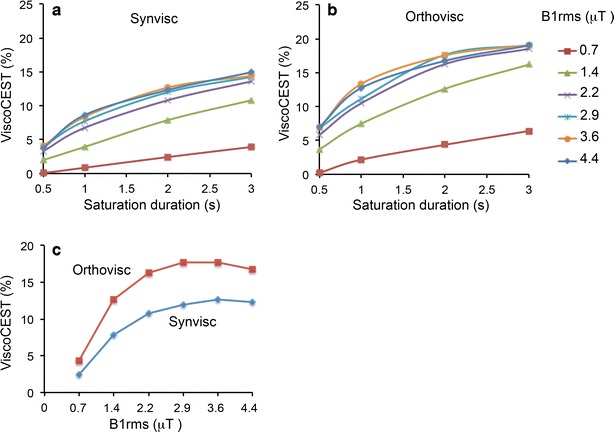
Saturation pulse and saturation duration dependence ViscoCEST contrast. **a** and **b** Graphs show the saturation pulse amplitude (B_1rms_) and saturation duration dependent ViscoCEST contrast from Synvisc and Orthovisc. Increase in ViscoCEST contrast is observed with increased saturation power and saturation pulse duration. **c** Graph is showing B_1rms_ dependent ViscoCEST contrast from Orthovisc and Synvisc for the saturation duration of 2 s

Figure 3 shows B_1rms_ and saturation duration dependent ViscoCEST effect from these molecules. Both viscosupplementations showed increased CEST contrast with increase in B_1rms_ and saturation pulse duration. Graphs clearly showed that the optimal B_1rms_ is 3.6 μT to observe the optimal ViscoCEST contrast from both viscosupplementations. The saturation parameters can be further optimized, however, in the current study, it was constrained by the SAR limit of the scanner.

Currently, there is no technique to monitor and track the fate of the injected viscosupplementations in knee joints of OA patients over time as well as their effect on knee cartilage GAG concentration. Different in vivo MR studies have been reported to monitor the changes in GAG content in human knee cartilage. The GdDTPA enhanced method [25–27] and spin lock T_1rho_ techniques [28, 29] have been widely used to monitor the GAG changes in knee cartilage. The beneficial effect of intra-articular injected HA in increasing the total cartilage volume in knee osteoarthritis patients who underwent high tibial osteotomy has been shown recently [30]. However, none of these methods can provide changes in the GAG concentration following injection of viscosupplementations. Recently, the GagCEST method has been developed to image the GAG concentration in cartilage. The GagCEST technique has been evaluated to map the GAG changes in knee cartilage both at 3T and 7T [20]. Anup et al. have showed that using GagCEST technique the knee GAG content can be imaged at high resolution on 7T, while no appreciable GagCEST contrast was observed from 3T [20]. More direct water saturation and fast exchange regime of the GAG –OH protons at 3T compared to 7T could be the possible reasons for the negligible GagCEST contrast at 3T.

**Fig. 4:**
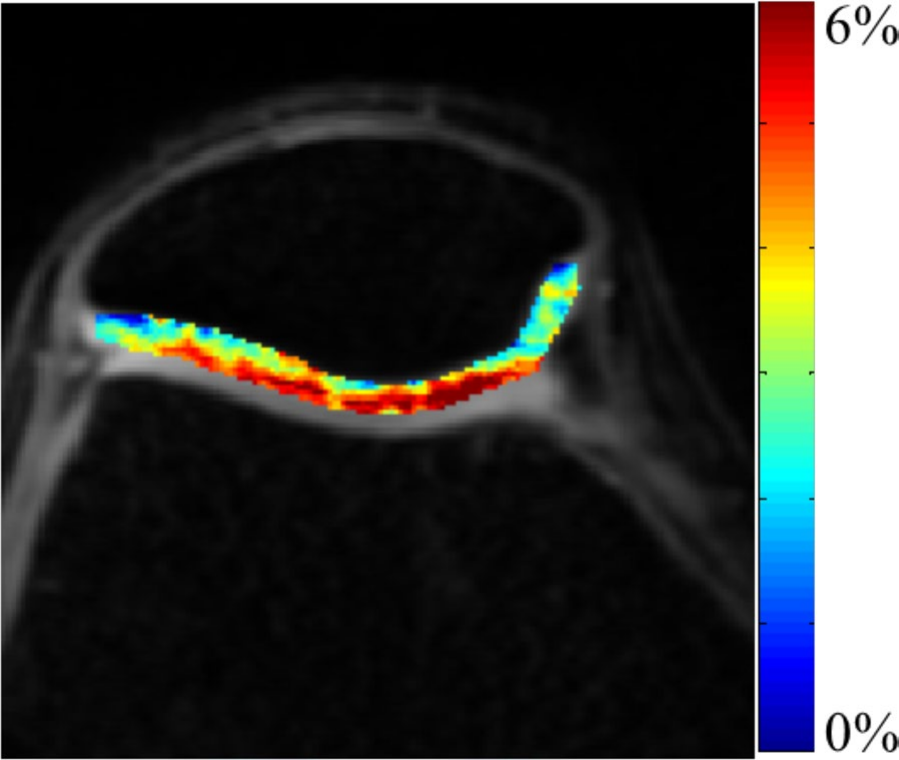
The GagCEST map from a healthy human knee cartilage is showing distribution of glycosaminoglycan on knee cartilage

Singh et al. have showed that the optimal parameter to observe the GagCEST contrast at 7T is 2.2 μT B_1rms_ and 500 ms saturation pulse duration [20]. We reproduced the GagCEST map of a human knee cartilage at 7T using the optimal GagCEST parameters as described by Singh et al. showed similar GagCEST contrast (Fig. 4). Increasing or decreasing saturation pulse power or saturation pulse duration leads to decrease in GagCEST contrast from the knee cartilage. While, the current study showed that 3.6 μT Hz is the optimal B_1rms_ and saturation duration of >1.5 s is required to observe optimal ViscoCEST contrast from both viscosupplementations. It should be noted that with optimal parameters for endogenous GagCEST (2.2 μT B_1rms_ and 500 ms saturation) both the viscosupplementations provide a ViscoCEST contrast of only ~30 % of the maximum value. On the other hand, with the optimal parameters for viscosupplmentation, the endogenous GagCEST from cartilage is negligibly small. Therefore, using GagCEST, it may be possible to monitor the GAG content changes in cartilage following viscosupplemntation injection, and at the same time ViscoCEST potentially enables probing of temporal and spatial distribution of viscosupplementations in the intraarticular space.

## Conclusions

Our initial results demonstrate that both Orthovisc than Synvisc viscosupplements exhibit –OH group exchange mediated CEST effect and they can be measured using ViscoCEST. In addition, ViscoCEST has potential to assess the spatial and temporal variation of these viscosupplementats in vivo. Further studies in these lines are currently in progress in our laboratory.

## Abbreviations

OA: osteoarthritis
HA: hyaluronic acid
MRI: magnetic resonance imaging
GAG: glycosaminoglycan
CEST: chemical exchange saturation transfer
JSN: joint space narrowing
MT: magnetization transfer
GRE: spoiled gradient echo
SAR: specific absorption rate
